# Social determinants influencing cervical cancer diagnosis: an ecological study

**DOI:** 10.1186/s12939-023-01912-8

**Published:** 2023-05-25

**Authors:** Juan Fernando Galindo, Giovana Moura Formigari, Luiz Carlos Zeferino, Carla Fabrine Carvalho, Edson Luiz Ursini, Diama Bhadra Vale

**Affiliations:** 1 School of Technology, University of Campinas, Rua Paschoal Marmo 1888, Limeira, 13484-332 Brazil; 2 Gynecology and Obstetrics Department, University of Campinas, Rua Vital Brazil 80, Campinas, 13083-888 Brazil

**Keywords:** Uterine Cervical Neoplasms, Social Determinants of Health, Social Vulnerability, Health Equity

## Abstract

**Background:**

Barriers to accessing health care result in advanced cervical cancer. In Sao Paulo, Brazil, the Index of Social Responsibility (ISR) synthesizes the situation of each town concerning wealth, education, and longevity. This study aimed to evaluate in 645 municipalities the relation of the ISR with stage, age, and morphology in cervical cancer diagnosis.

**Methods:**

An ecological study that used data from Sao Paulo, Brazil, from 2010 to 2017. The ISR was identified through government platforms and data on cancer through the Hospital Cancer Registry. The subjects were the 9,095 women aged 30 years or older. The ISR summarizes municipalities into five levels: dynamic (ISR5), unequal (ISR4), equitable (ISR3), in transition (ISR2), and vulnerable (ISR1). It was used the *chi*^2^ tests and logistic regression.

**Results:**

The proportion of stage 1 increased significantly with ISR level, ranging from 24.9% in ISR1 to 30.0% in ISR5 (p = 0.040). To every increase in ISR level, the chance of a woman being diagnosed in stage I was at least 30% higher. Woman living where ISR2 had a 1.4 times higher chance of being diagnosed in stage 1 than those living in ISR1 (OR 1.40, 95% CI 1.07–1.84). Squamous tumors frequency decreased when ISR level increased (p = 0.117). A higher proportion of women under 50 years were observed when they lived in wealthier cities (ISR4 and ISR5) (42.2% vs. 44.6%, p = 0.016).

**Conclusion:**

The ISR was a good health indicator for understanding and predicting the social determinants in cervical cancer diagnosis. The proportion of stage I increased significantly in more favorable social conditions.

## Background

Cervical cancer is a significant cause of death among women worldwide, and its occurrence is associated with the development of countries and their regions [1]. Vaccine and screening are the main strategies to prevent this disease [2, 3]. Only 21% of cases in Brazil are diagnosed in stage I, which includes those diagnosed through screening [4]. Brazillian states with a higher Human Development Index present more cases at early stages and a higher density of hospitals and physicians for cancer diagnosis and treatment [4].

Social determinants of health are the non-medical factors that influence health outcomes [5]. It is essential to consider their influence on all spheres of the health-disease process, at the individual and collective levels. Cervical cancer is possibly prevented. A woman diagnosed in advanced stages probably was influenced by social determinants of health, individually or collectively [2, 4, 6–9]. It is necessary to identify and analyze how this interaction impacts the proportion of women diagnosed in advanced stages.

The Index of Social Responsibility (ISR) is an indicator used to support municipalities in planning public policies in Sao Paulo state, Brazil, since 2000. The ISR synthesizes the situation of each town concerning wealth, education, and longevity. It categorizes municipalities into five levels: dynamic, unequal, equitable, in transition, and vulnerable [10]. The hypothesis is that a lower ISR level will be associated with poor diagnosis status and other variables related to access to health care.

This study aims to evaluate the relations of the ISR of Sao Paulo’s municipalities with stage, age, and morphology in cervical cancer diagnosis. A better understanding of how social determinants impact the diagnosis can help managers define public cancer prevention policies to improve women’s lives and communities.

## Methods

This ecological study related the Index of Social Responsibility (ISR) with variables of cervical cancer diagnosis in Sao Paulo State, from 2010 to 2017. The ISR was identified through the ‘Fundação Seade’ and the Legislative Assembly of the State of Sao Paulo [10]. Data on cancer diagnosis was accessed from the Hospital Cancer Registry of Sao Paulo (HCR-SP) [11]. All data is available for the general public on online platforms.

The subjects were the 9,502 women aged 30 years or older registered at the HCR-SP from 2010 to 2017, by the code C-53 (cervical cancer), according to the International Classification of Diseases, 10th edition (ICD-10). Women under 30 were excluded because they present a more variable diagnostic profile, carrying more aggressive tumors [12, 13]. Those with an unknown stage (n = 407) were also excluded. The final sample consisted of 9,095 cases. At HCR-SP, data is anonymous and entered into an online platform by trained hospital technicians. All hospitals licensed for cancer care in Sao Paulo must provide data to the HCR-SP. Only cases registered for primary treatment were included. Data completeness is high [4, 13, 14]. In this sample, the stage was absent in 4.3%, the morphology was categorized as “ill-defined” in 5.2%, and no age information was missing.

The outcomes were age, stage, and morphology. For analysis, the following categories were created: women younger than 50 years old (< 50 years old) and 50 years old or older (≥ 50 years old); stage 1 and stage 2+ (2 or more severe), according to FIGO 2009 [15]; and tumors with squamous morphology (SCC) or others (adenocarcinomas, adenosquamous carcinomas, and other types). Stage 1 represents cases detected by screening, and stage 2 + represents cases diagnosed symptomatically – not submitted due to prevention practices. It is an indicator of the healthcare system’s fragility [2, 4]. Oldest ages at diagnosis are expected to be related to barriers to access to care and knowledge, as younger women visit more health services and are exposed to more information in their communities [14, 16]. Regarding morphology, the frequency of adenocarcinomas is higher in places where screening practices are more effective, as squamous morphology is more commonly diagnosed through screening [17].

The ISR summarizes the situation of each of the 645 Sao Paulo’s municipalities regarding wealth, education, and longevity. Wealth is composed of Gross Domestic Product per capita; Remuneration of formal employees; Consumption of electricity in residences, agriculture, trades, and others. Education is the proportion of students in public schools with an adequate level in Portuguese (native language) and mathematics, and in the 5th and 9th year of teaching elementary; school attendance rate, age-grade distortion rate in the high school. Longevity is perinatal and infant mortality; mortality from 15 to 39 years old; and 60 to 69 years old.

When these dimensions are combined, they generate a typology that classifies municipalities into five levels [10]: *Dynamic*, those with a high level of wealth and social indicators (ISR5); *Unequal*, those that, despite having high levels of wealth, do not achieve good indicators in social dimensions (ISR4); *Equitable*, those with low levels of wealth, but good social indicators (ISR3); *In transition*, cities with low levels of wealth and intermediate levels of longevity and/or education (ISR2); and *Vulnerable*, the most disadvantaged, both in terms of wealth and social indicators (ISR1).

Data were available for the years 2014, 2016, and 2018. We chose the mode of the three years for this analysis, except when there were three different values when the median was chosen. Since the HCR-SP data covers an extended period (2010 to 2017), it was necessary to weigh the ISR values of each city. For a more accurate sub-analysis of the indicators, ISR sub-groups were created according to their components: lower wealth (ISR 1 to 3) and higher wealth (ISR 4 and 5); deep inequities (ISR 1 and 2), and less inequity (ISR 3). We also performed an analysis for each level increase to determine which level increase would be more sensible for each outcome.

For statistical analysis, it was used the *chi*^2^ tests and logistic regression with the calculation of odds ratios (OR), assuming a significance level of 5% (p < 0.05) and a 95% confidence interval (CI). This study is part of the research ‘Factors Related to Late Diagnosis of Cervical Cancer’ that develops a model to predict advanced stages in cervical cancer. The project was approved by the ‘Ethics and Research Committee of Unicamp’, under the number CAAE: 42657020.1.0000.5404. The Committee waived the need for informed consent due to the secondary data nature of the study. The data used are public and available online on official computer platforms of the State of Sao Paulo government. The women’s identification could not be accessed.

## Results

**Fig. 1:**
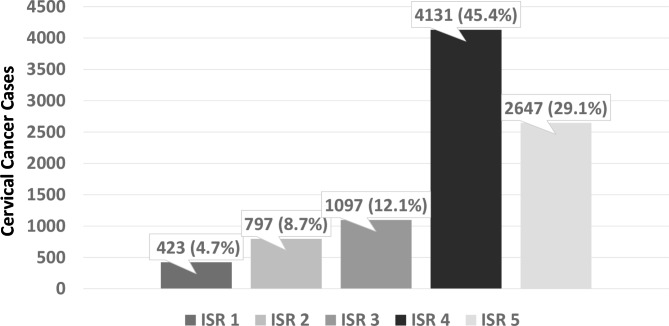
Distribution of 9,095 cases of cervical cancer according to the Index of Social Responsibility of the State of Sao Paulo, Brazil, from 2010 to 2017

From 2010 to 2017 9,095 cervical cancer cases in women aged 30 years or older were registered in the HCR-SP. The distribution of cases according to the ISR can be seen in Fig. 1. Most cases came from cities with ISR4 (45.4%) and ISR5 (29.1%).

Table 1 shows the distribution of cervical cancer cases among the five ISR levels as a function of the stage, age, and morphology groups. It was observed that the proportion of stage 1 (early stage) increased significantly with the ISR, ranging from 24.9% in ISR1 to 30.0% in ISR5 (p = 0.040). There was no variation in the age group as a function of the ISR: the proportion of cases in women aged 50 years or older ranged from 54.0 to 60.2% (p = 0.117). Squamous tumors were the most frequent, and their proportion decreased when related to the ISR (p = 0.117).

**Table 1:** Distribution of cervical cancer cases by the vulnerable indicator ISR where the women live, according to stage, age-group, and morphology in Sao Paulo, Brazil, 2010 to 2017

	ISR1	ISR2	ISR3	ISR4	ISR5	
	n (%)	n (%)	n (%)	n (%)	n (%)	P-value
**Total**	354	839	1101	4137	2664	
	**Stage**					
**Stage 1**	88 (24.9)	233 (27.8)	305 (27.7)	1209 (29.2)	798 (30.0)	**0.040**
**Stage 2+**	266 (75.1)	606 (72.2)	796 (72.3)	2928 (70.8)	1866 (70,0)	
	**Age-group**					
**< 50 years**	152 (42.9)	334 (39.8)	481 (43.7)	1904 (46.0)	1132 (42.5)	0.117
**≥ 50 years**	202 (57.1)	505 (60.2)	620 (56.3)	2233 (54.0)	1532 (57.5)	
	**Morphology**					
**SCC**	264 (74.6)	628 (74.9)	799 (72.6)	3113 (75.3)	1780 (66.8)	0.117
**Others**	90 (25.4)	211 (25.1)	302 (27.4)	1024 (24.7)	884 (33.2)	

*ISR – Index of Social Responsibility; P-value - chi* ^*2*^ *test; Stage 2+ - Stages II, III, and IV; SCC – Squamous cell carcinoma.*

For each ISR level increase, the chance of presenting cervical cancer in stage I was at least 30% higher than in the previous ISR. In the multivariate analysis, the risk was more significant when comparing ISR1 and ISR2: women living where ISR2 had a 1.4 times higher chance of being diagnosed in stage 1 than those living where ISR1 (OR 1.40, 95% CI 1.07–1.84). No variations in risk were observed regarding the age group. Regarding morphology, a 31% lower risk of having a squamous tumor was observed in those women living in ISR5 than ISR4 (OR 0.69, 95% CI 0.55–0.86) (Table 2).

**Table 2:** Risks in cervical cancer cases by the ISR where the women live, according to stage, age-group, and morphology in Sao Paulo, Brazil, 2010 to 2017

			Univariate analysis			Multivariate analysis		
**Stage**								
**ISR**	**1**	**2+**	**P**	**OR**	**95% CI**	**P**	**OR**	**95% CI**
**1**	88	266	-	-	-	-	-	-
**1 vs. 2**	233	606	0.025	1.36	1.04–1.77	**0.016**	1.40	1.07–1.84
**2 vs. 3**	305	796	0.027	1.33	1.04–1.71	**0.037**	1.31	1.02–1.70
**3 vs. 4**	1209	2928	0.006	1.37	1.10–1.72	**0.011**	1.35	1.07–1.70
**4 vs. 5**	798	1866	0.002	1.43	1.14–1.80	**0.006**	1.39	1.10–1.76
**Age-group**								
**ISR**	**< 50 y**	**≥ 50 y**	**P**	**OR**	**95% CI**	**P**	**OR**	**95% CI**
**1**	152	202	-	-	-	-	-	-
**1 vs. 2**	334	505	0.506	1.08	0.86–1.36	0.272	1.14	0.90–1.45
**2 vs. 3**	481	620	0.730	0.96	0.78–1.19	0.970	1.00	0.81–1.25
**3 vs. 4**	1904	2233	0.075	0.84	0.69–1.02	0.205	0.88	0.73–1.07
**4 vs. 5**	1132	1532	0.918	0.99	0.81–1.20	0.715	1.04	0.85–1.27
**Morphology**								
**ISR**	**SCC**	**Others**	**P**	**OR**	**95% CI**	**P**	**OR**	**95% CI**
**1**	264	90	-	-	-	-	-	-
**1 vs. 2**	628	211	0.594	0.96	0.74–1.26	0.990	1.00	0.76–1.30
**2 vs. 3**	799	302	0.454	0.88	0.69–1.12	0.412	0.90	0.70–1.15
**3 vs. 4**	3113	1024	0.890	1.02	0.82–1.27	0.677	1.05	0.84–1.30
**4 vs. 5**	1780	884	0.001	0.67	0.53–0.83	**0.001**	0.69	0.55–0.86

*ISR – Index of Social Responsibility; Stage 2+ - Stages II, III and IV; SCC – Squamous cell carcinoma; P – P-value by Logistic regression; OR – Odds Ratio; 95% CI – 95% Confidence Interval.*

X vs. Y means one group of ISR compared to the next one.

Table 3 shows the distribution of cervical cancer cases in the ISR levels according to the wealth component, split into lower wealth (ISR1, ISR 2, and ISR3) and higher wealth (ISR4 and ISR5). A higher proportion of women under 50 years was observed when they lived in wealthier cities (42.2% vs. 44.6%, p = 0.016). In the risk analysis (Table 4), the chance of a cervical cancer case in a woman aged 50 years or older was 10% lower when they lived where there was a higher level of wealth (ISR4 and ISR5) (OR 0.90, 0.82–0.99).

**Table 3:** Distribution of cervical cancer cases by groups of wealth from the ISR where the women live, according to stage, age-group, and morphology in Sao Paulo, Brazil, 2010 to 2017

	ISR1, ISR2, ISR3	ISR4, ISR5	
	n (%)	n (%)	P-value
**Total**	2294 (100)	6801 (100)	
	**Stage**		
**Stage 1**	626 (27.3)	2007 (29.5)	0.067
**Stage 2+**	1668 (72.7)	4794 (70.5)	
	**Age-group**		
**< 50 years**	967 (42.2)	3036 (44.6)	0.016
**≥ 50 years**	1327 (57.8)	3765 (55.4)	
	**Morphology**		
**SCC**	1691 (73.7)	4893 (72.0)	0.1080
**Others**	603 (26.3)	1908 (28.0)	

*ISR – Index of Social Responsibility; P-value - chi2 test; Stage 2+ - Stages II, III and IV; SCC – Squamous cell carcinoma.*

**Table 4:** Risks in cervical cancer cases by groups of wealth from the ISR where the women live, according to stage, age-group, and morphology in São Paulo, Brazil, 2010 to 2017

			Univariate analysis			Multivariate analysis		
**1–3**	626	1668	-	-	-	-	-	-
**4–5**	2007	4794	0.068	1.10	0.99–1.23	0.202	1.07	0.96–1.19
**Age-group**								
**ISR**	**< 50 y**	**≥ 50 y**	**P**	**OR**	**95% CI**	**P**	**OR**	**95% CI**
**1–3**	967	1327	-	-	-	-	-	-
**4–5**	3036	3765	**0.018**	0.89	0.81–0.98	**0.034**	0.90	0.82–0.99
**Morphology**								
**ISR**	**SCC**	**Others**	**P**	**OR**	**95% CI**	**P**	**OR**	**95% CI**
**1–3**	1691	603	-	-	-	-	-	-
**4–5**	4893	1908	0.118	0.92	0.83–1.02	0.145	0.92	0.83–1.03

*ISR – Index of Social Responsibility; Stage 2+ - Stages II, III, and IV; SCC – Squamous cell carcinoma; P – P-value by Logistic Regression; OR – Odds Ratio; 95% CI – 95% Confidence Interval.*

X vs. Y means one group of ISR compared to the next one.

Table 5 shows the distribution of cervical cancer cases in the ISR levels according to the inequity component, split into more inequity (ISR1 and ISR2) and less inequity (ISR 3). There was no difference in the proportion of cases in any of the variables analyzed. No significant difference was observed in the risk analysis (Table 6).

**Table 5:** Distribution of cervical cancer cases by groups of inequity from ISR where the women live, according to stage, age-group, and morphology in Sao Paulo, Brazil, 2010 to 2017

	ISR1, ISR2	ISR3	
	n (%)	n (%)	P-value
**Total**	1193 (100)	1101 (100)	
	**Stage**		
**Stage 1**	321 (26.9)	305 (27.7)	0.255
**Stage 2+**	872 (73.1)	796 (72.3)	
	**Age-group**		
**< 50 years**	486 (40.7)	481 (43.7)	0.262
**≥ 50 years**	707 (59.3)	620 (56.3)	
	**Morphology**		
**SCC**	892 (74.8)	799 (72.6)	0.312
**Others**	301 (25.2)	302 (27.4)	

*ISR – Index of Social Responsibility; P-value - chi2 test; Stage 2+ - Stages II, III and IV; SCC – Squamous cell carcinoma.*

**Table 6:** Risks in cervical cancer cases by groups of inequity from the ISR where the women live, according to stage, age-group and morphology in Sao Paulo, Brazil, 2010 to 2017

			Univariate analysis			Multivariate analysis		
**Stage**								
**ISR**	**1**	**2+**	**P**	**OR**	**95% CI**	**P**	**OR**	**95% CI**
**1–2**	321	872	-	-	-	-	-	-
**3**	305	796	0.255	0.90	0.75–1.08	0.504	1.07	0.88–1.28
**Age-group**								
**ISR**	**< 50 y**	**≥ 50 y**	**P**	**OR**	**95% CI**	**P**	**OR**	**95% CI**
**1–2**	486	707	-	-	-	-	-	-
**3**	481	620	0.303	0.92	1.17–1.47	0.396	0.93	0.79–1.10
**Morphology**								
**ISR**	**SCC**	**Others**	**P**	**OR**	**95% CI**	**P**	**OR**	**95% CI**
**1–2**	892	301	-	-	-	-	-	-
**3**	799	302	0.255	0.90	0.75–1.08	0.293	0.91	0.75–1.09

*ISR – Index of Social Responsibility; Stage 2+ - Stages II, III, and IV; SCC – Squamous cell carcinoma; P – P-value by Logistic Regression; OR – Odds Ratio; 95% CI – 95% Confidence Interval.*

X vs. Y means one group of ISR compared to the next one.

## Discussion

In this study, the ISR was presented as a good health indicator for understanding and predicting social determinants in cervical cancer diagnosis. The proportion of an early stage at diagnosis increased significantly due to the elevation of the ISR level. ISR discreetly influences age at diagnosis.

For each ISR level increase, the chance of presenting a stage I diagnosis was at least 30% higher than the previous. The hypothesis is that cities with higher ISR have a better health system framework, allowing women to have better screening and early diagnosis access. Screening practices are complex health activities, as they involve, in addition to testing the target population, the assessment and treatment of positive cases. It requires articulating different levels of care. Quality control at all steps of the process is essential to ensure effectiveness. In the literature, there is solid evidence that cervical cancer incidence is related to the region’s level of development or to the health system’s ability to diagnose and treat precursor lesions [2, 18, 19]. Higher the development of a country or region, earlier the stage at diagnosis [4, 14, 20, 21].

In our study, the proportion of cases in women aged 50 years and over ranged from 54.0 to 60.2% (p = 0.117). A higher proportion of cases in women under 50 was found in the wealthier cities (42.2% vs. 44.6%, p = 0.016). Younger women have more opportunities to access the health system through prenatal care, menstrual complaints, contraception, and others. In cities with better wealth indicators, there may have been an anticipation of the diagnosis by providing more essential services to the population. Other studies point out that young women present more early stage cervical cancer than the older [14, 16, 22, 23].

Squamous tumors were the most frequent, and their proportion decreased through the ISR levels, although not significantly (p = 0.117). The risk of presenting a squamous histology tumor was 31% lower than non-squamous histology in ISR5 than ISR4. Squamous tumors are the most frequently diagnosed through screening [17]. With improvements in screening, the proportion of squamous tumors decreases. In Norway, a study reported a 30% reduction in squamous cell tumors and a 38% increase in adenocarcinomas from 1970 to 1984 [24]. Studies carried out in Sweden, Canada and England showed similar trends in their populations [25–27].

Low and middle-income countries present most cervical cancer cases and many health inequalities [1, 19]. At the sub-analysis of the ‘inequality’ component, no difference was observed in the proportion of cases in any variable used. If the detection of cases at an early stage depends on a complex health services framework, the extremes of vulnerability seem to have little influence on cancer diagnosis. A minimum of development is necessary to observe the impacts of the actions. In this study, differences were observed only at the extremes of wellness. Reducing the inequalities seems to be the more complex challenge in cervical cancer control in Brazil.

In this study, the ISR was presented as a good health indicator for understanding and predicting social determinants in cervical cancer diagnosis. Among the components of the ISR, ‘wealth’ seemed to be the most sensitive, reinforcing the hypothesis that a certain degree of infrastructure in health is necessary so that the impact of cervical cancer prevention actions to be observed. The ISR gathers education, income, and longevity, grouping São Paulo municipalities according to their degree of vulnerability. In this study, greater degrees of vulnerability in the municipalities were more related to cervical cancer diagnosis at a more unfavorable profile.

The strength of this study lies in the detailing of data from a large sample of cities, allowing for significant ecological inferences. The main limitation that may have confounded the results is that 28.5% of the cases belong to the city of Sao Paulo, where most of the health services are concentrated, a city with more than 10 million inhabitants and significant variability in living conditions. It is also noticeable that the ISR is based in the context of the state of Sao Paulo. The fact that there is a significant relationship between the stage of the disease and the ISR highlights the strength of a multidimensional index for decision-making processes in public health. The merged dataset may be helpful for these purposes and further analyses related to cervical cancer and social determinants of health.

## Conclusions

The ISR was a good health indicator for understanding and predicting how social determinants impacts cervical cancer diagnosis. The proportion of stage I increased significantly due to the increase of ISR level, although age has discreetly influenced it. Women’s better access to screening and early diagnosis may justify this relationship.

## Data Availability

Deidentified participant data and data on the Index of Social responsibility are open to the general public through the following websites in Portuguese (access on Mar 13, 2022): http://www.fosp.saude.sp.gov.br/fosp/diretoria-adjunta-de-informacao-e-epidemiologia/rhc-registro-hospitalar-de-cancer/banco-de-dados-do-rhc/. http://catalogo.governoaberto.sp.gov.br/dataset/20-indice-paulista-de-responsabilidade-social-iprs. Additional documents, including study protocol, Ethic’s Committee approval, crude tables, and statistical analysis documents, are available under reasonable requests. The authors will be glad to share data to support new studies or further analysis.

## Abbreviations

< 50: Women younger than 50 years old
≥50: Women 50 years old or older
CI: 95% confidence interval
HCR-SP: Hospital Cancer Registry of Sao Paulo State
ICD-10: International Classification of Diseases, 10th edition
ISR: Index of Social Responsibility
ISR1: Vulnerable municipality - disadvantaged, both in terms of wealth and social indicators
ISR2: In transition municipality - low levels of wealth and intermediate levels of longevity and/or education
ISR3: Equitable municipality - low levels of wealth but good social indicators
ISR4: Unequal municipality - high levels of wealth but does not achieve good indicators in social dimensions
ISR5: Dynamic municipality - high level of wealth and social indicators
OR: Odds ratios
1-p: Probability of failure
p: Probability of success
p < 0.05: a significance level of 5%
SCC: squamous cell carcinoma
Stage 2+: Stage 2 or more severe
